# One-Year Follow-Up of Red Yeast Rice-Associated Renal Dysfunction: A Report of Two Cases

**DOI:** 10.7759/cureus.101317

**Published:** 2026-01-11

**Authors:** Moe Ozawa, Yuki Kawai, Jin Oshikawa, Kiyotaka Nagahama, Tamio Iwamoto, Kouichi Tamura

**Affiliations:** 1 Department of Nephrology and Hypertension, Saiseikai Yokohamashi Nanbu Hospital, Yokohama, JPN; 2 Department of Nephrology, Yokohama Sakae Kyosai Hospital, Yokohama, JPN; 3 Department of Pathology, Kyorin University School of Medicine, Tokyo, JPN; 4 Department of Nephrology and Hypertension, Yokohama City University Medical Center, Yokohama, JPN

**Keywords:** beni-koji, fanconi syndrome, follow-up appointment, red yeast rice, renal dysfunction

## Abstract

Red yeast rice is used for treating hyperlipidemia because it lowers low-density lipoprotein-cholesterol concentrations. However, red yeast rice supplements (Beni-koji tablets) were recalled nationwide in Japan in 2024 following the identification of multiple cases of renal dysfunction associated with specific batches. Renal dysfunction associated with Beni-koji tablets has been reported to result in Fanconi syndrome accompanied by a reduction in estimated glomerular filtration rate. The primary means of management is to discontinue the tablets, but few studies have examined the long-term renal outcomes. We report two cases of renal dysfunction followed for one year after the discontinuation of the Beni-koji tablets. Case 1 was a 60-year-old woman who presented with severe renal dysfunction and Fanconi syndrome after taking Beni-koji tablets. Her renal function partially recovered after discontinuation of the tablets and remained stable during follow-up. Case 2 was a 56-year-old woman who exhibited moderate-to-severe renal dysfunction and Fanconi syndrome after prolonged intake of Beni-koji tablets. Following corticosteroid therapy, in addition to discontinuation of the tablets, her renal function fully recovered and remained stable thereafter. These cases suggest that renal function may recover gradually after discontinuation of Beni-koji tablets. Careful follow-up for at least six months is advisable, as renal recovery tends to stabilize after this period, to confirm sustained improvement and monitor for residual tubular dysfunction.

## Introduction

Red yeast rice (RYR) is a product of *Monascus* fungi grown on rice. Traditionally, it has been used in Asia for producing fermented foods and as a food coloring agent. Recently, RYR has also been used as a functional food for managing hyperlipidemia in Japan, as it lowers circulating low-density lipoprotein-cholesterol concentrations. However, reports of renal dysfunction associated with specific batches of RYR supplements (Beni-koji tablets) were received in Japan from late 2023, prompting a recall by Kobayashi Pharmaceuticals in March 2024 [1].

A nationwide survey of 192 patients was conducted through physicians who were members of the Japanese Society of Nephrology [2]. The survey revealed that most patients developed Fanconi syndrome, which is characterized by global dysfunction of proximal tubular reabsorption leading to excessive loss of solutes, including phosphate, uric acid, glucose, low-molecular-weight proteins, amino acids, and bicarbonate. These patients also exhibited decreased estimated glomerular filtration rate (eGFR; <60 mL/minute/1.73 m²). In this survey, renal function was monitored in most cases for several weeks to several months, and the primary intervention was the discontinuation of the Beni-koji tablets. Because observation periods were relatively short, the long-term course of renal recovery remains unclear. We describe two cases with detailed monitoring of renal function changes over at least the first year following tablet discontinuation, providing insight into the observation period of renal recovery. These two cases were not consecutive; the patients were seen at different affiliated hospitals within the same university network.

## Case presentation

Case 1

Case 1 was a 60-year-old woman with a history of gastroesophageal reflux but no renal disease. Her serum creatinine concentration was 0.74 mg/dL five months before taking Beni-koji tablets (Beni Koji Choleste Help, Kobayashi Pharmaceutical Co., Ltd., Osaka, Japan). She had no history of hypertension, diabetes, or obesity. After consuming the tablets for four months, she developed a loss of appetite and visited a local internal medicine clinic, where abdominal CT and upper gastrointestinal endoscopy showed no abnormalities. Two months later, she visited another local internal medicine clinic because she had noticed foamy urine, which suggested proteinuria, and had lost 5 kg of body mass. She was then referred to our hospital in January 2024 because of proteinuria, hematuria, and glucosuria.

At presentation (Day zero), her vital signs were within normal limits. Table 1 shows the results of laboratory tests. Urinalysis revealed hematuria, renal glucosuria, and proteinuria, along with markedly elevated markers of renal tubular dysfunction. Blood testing revealed a high serum creatinine concentration (1.87 mg/dL), and, therefore, a low eGFR (22 mL/minute/1.73 m²), as well as hypouricemia, hypophosphatemia, and euglycemia. Therefore, findings were consistent with acute kidney injury associated with Fanconi syndrome due to proximal tubular disorders. Other causes of rapidly progressive renal dysfunction, including autoimmune diseases (Table 2) and urinary tract obstruction on abdominal CT, were excluded. Furthermore, potential causes of acquired Fanconi syndrome were ruled out. However, given the temporal association with Beni-koji tablets, discontinuation was advised at her first visit.

**Table 1:** Laboratory findings of Case 1 and Case 2. Urinalysis and blood test results for Case 1 and Case 2 at presentation, six months, and the latest follow-up, with reference ranges. β2MG = β2-microglobulin; NAG = N-acetylglucosaminidase; BUN = blood urea nitrogen; Cr = creatinine; eGFR = estimated glomerular filtration rate; UA = uric acid; Na = sodium; K = potassium; Cl = chlorine; Ca = calcium; iP = inorganic phosphate; NA = not available; Glu = glucose; HCO_3^−^_ = bicarbonate

Variable			Case 1			Case 2		
Age (years)			60			56		
Sex			Female			Female		
Duration of Beni-koji tablet intake			6 months			1 year		
		Reference range	At first visit (Day 0)	After 6 months (Day 161)	Latest (Day 413)	At first visit (Day 0)	After 6 months (Day 191)	Latest (Day 443)
Urinalysis	Specific gravity	-	1.017	1.008	1.015	1.027	1.013	1.019
	pH	-	6.0	6.0	5.0	6.5	7.5	6.0
	Protein	-	2+	-	-	2+	-	-
	Glucose	-	4+	-	-	3+	-	-
	Occult blood	-	2+	-	+/–	1+	-	-
	Urine protein (g/gCr)	-	2.34	Undetectable	0.06	2.34	0.04	0.04
	β2MG (µg/L)	0–289	15,900	152	21	82,520	400	580
	NAG (U/L)	0.7–11.2	30.8	0.7	6.8	26.7	2.0	3.1
Blood chemistry	BUN (mg/dL)	8–20	30.1	19.5	20.5	16.6	12.1	13.1
	Cr (mg/dL)	0.46–0.79	1.87	0.95	0.87	1.39	0.77	0.73
	eGFR (mL/minute/1.73m²)	-	22	47	51	31	60	63
	UA (mg/dL)	2.6–5.5	1.6	4.3	5.1	1.4	4.4	4.6
	Na (mEq/L)	138–145	142	141	143	139	141	142
	K (mEq/L)	3.6–4.8	3.7	4.1	4.7	3.2	4.1	4.3
	Cl (mEq/L)	101–108	110	105	102	115	105	106
	Ca (mg/dL)	8.8–10.1	9.4	9.2	9.5	8.8	9.5	9.7
	iP (mg/dL)	2.7–4.6	1.9	4.0	3.3	1.4	3.8	3.4
	Glu (mg/dL)	73–109 (fasting blood glucose)	99	95	148	89	103	107
Blood gas (venous)	pH	-	NA	NA	NA	7.276	7.362	7.352
	HCO_3_^−^ (mEq/L)	21.2–27.0	NA	NA	NA	15.6	27.8	26.5
	CO_2_ (mmHg)	-	NA	NA	NA	34.6	50.2	48.9
	Base excess (mEq/L)	–3.2–1.8	NA	NA	NA	−10.0	2.1	0.8

**Table 2:** Laboratory results of Case 1. Immunoserological parameters for Case 1 with reference ranges. Ig = immunoglobulin; ANA = anti-nuclear antibody; MPO = myeloperoxidase; ANCA = anti-neutrophil cytoplasmic antibody

Immunoserological test	Reference range	At first visit (Day 0)
IgG (mg/dL)	1,155–1,723	954
IgA (mg/dL)	167–331	96
IgM (mg/dL)	136–256	68
C3 (mg/dL)	65–135	101
C4 (mg/dL)	13–35	30
ANA (×)	<40	<40
Anti-SS-A antibody (U/mL)	≤10	<1.0
Anti-SS-B-antibody (U/mL)	≤10	<1.0
MPO-ANCA (U/mL)	≤3.5	<1.0

**Figure 1:**
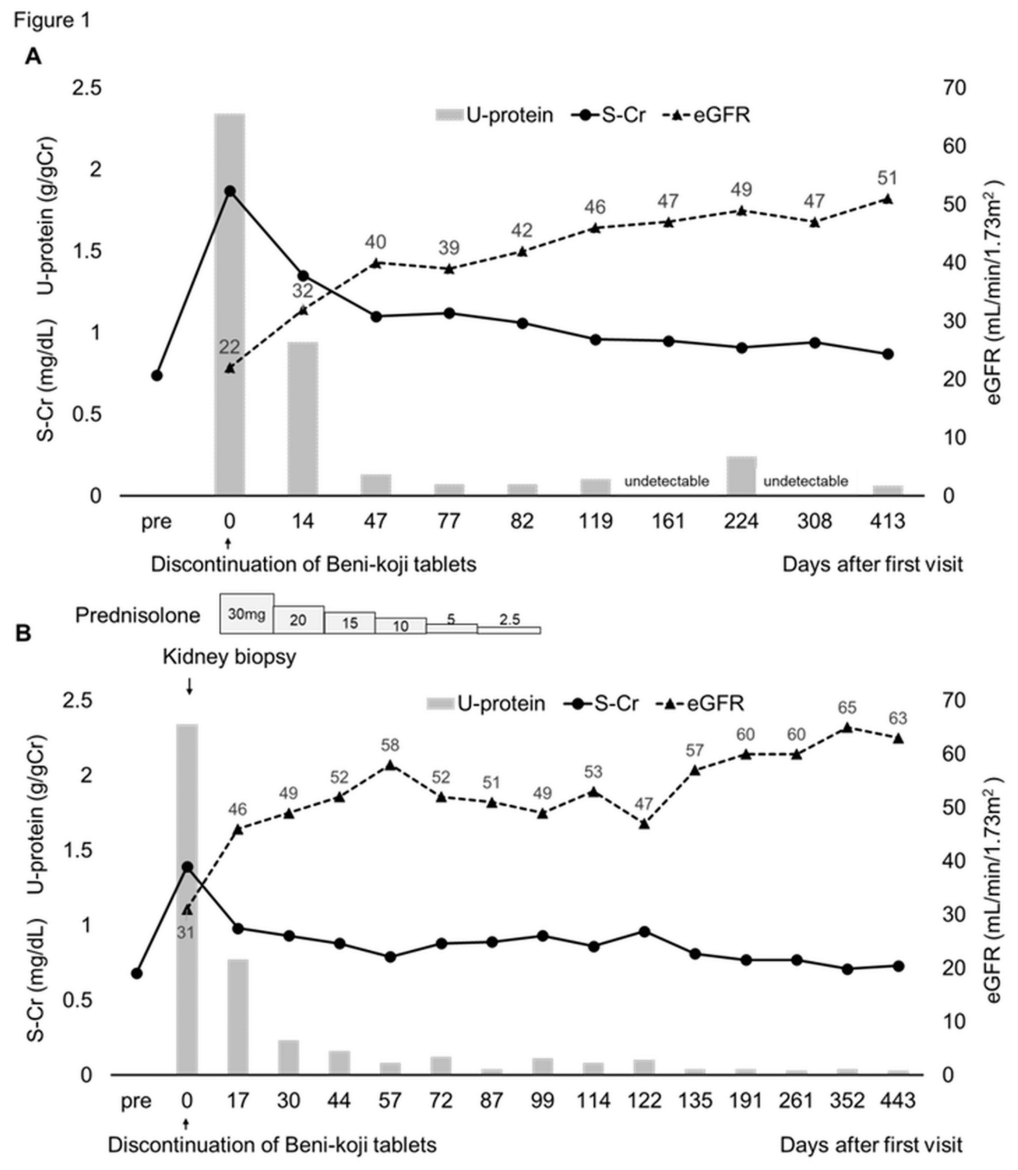
Clinical courses of two patients who developed renal dysfunction after taking Beni-koji tablets. Panels (A) and (B) show the clinical courses for Cases 1 and 2, including changes in estimated glomerular filtration rate (eGFR), serum creatinine (S-Cr), and urinary protein (U-protein).

Figure 1A and Figure 1B illustrate the clinical courses of Case 1 and Case 2, respectively. Two months after the discontinuation of the Beni-koji tablets, the electrolyte abnormalities and most urinalysis parameters gradually improved to within normal ranges. The markers of renal tubular dysfunction also returned to normal within approximately six months, whereas her eGFR exhibited partial recovery, remaining at approximately 50 mL/minute/1.73 m² after Day 119 (Table 1, Figure 1A). At her most recent follow-up appointment (on Day 413), her renal function remained stable, with an eGFR of 51 mL/minute/1.73 m². Urinalysis showed no recurrence of the proteinuria or glucosuria, and her renal tubular marker levels remained within the normal ranges. We did not perform a kidney biopsy as her renal function improved gradually following the discontinuation of the Beni-koji tablets.

Case 2

We previously reported the early clinical course of Case 2 [3]. Briefly, one year after consuming Beni-koji tablets, she was referred to our hospital in December 2023 because of renal dysfunction. She had no history of hypertension, diabetes, or obesity. She exhibited a high serum creatinine concentration (1.39 mg/dL), a low eGFR (31 mL/minute/1.73 m²), and Fanconi syndrome at presentation (Day zero) (Table 1).

**Figure 2:**
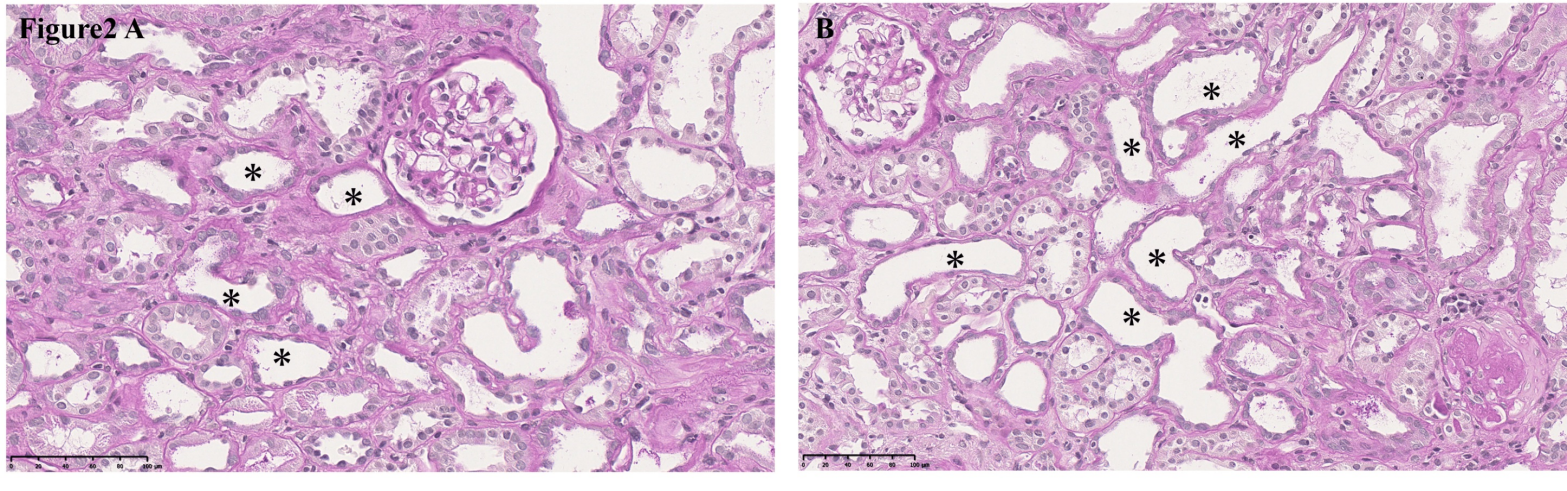
High-power view of periodic acid-Schiff staining of renal tissue demonstrating loss of brush border and simplification of the proximal tubular epithelium (asterisks) (scale bar = 100 μm).

Renal biopsy revealed acute proximal tubular injury (Figures 2A, 2B). The treatment included discontinuation of the Beni-koji tablets, correction of electrolyte imbalances, and corticosteroid therapy to suppress interstitial fibrosis. Within three months of tablet discontinuation, the electrolyte abnormalities had improved, and most urinalysis parameters had normalized. Her eGFR continued to improve until Day 191, returning to baseline levels (≥60 mL/minute/1.73 m²), and remained stable up to one year after discontinuation (Table 1, Figure 1B). At her most recent follow-up appointment (on Day 443), her renal function remained stable: she had an eGFR of 63 mL/minute/1.73 m². Her urinalysis findings, including the urinary protein and glucose levels, remained normal, and her renal tubular marker levels were quite stable throughout the follow-up period.

## Discussion

We reported the clinical course of two patients who developed renal dysfunction associated with the use of Beni-koji tablets, with renal function monitored for more than one year. The first patient’s eGFR gradually improved following discontinuation of the tablets, but remained below normal levels (approximately 50 mL/minute/1.73 m²) throughout the one-year follow-up period. In contrast, the second patient’s eGFR improved after discontinuation of the tablets, accompanied by corticosteroid therapy, and reached normal levels (≥60 mL/minute/1.73 m²), remaining stable thereafter.

The present findings suggest the importance of long-term follow-up because the recovery of renal function may take more than six months after the discontinuation of Beni-koji tablets. This is consistent with the findings of previous studies that assessed the effectiveness of corticosteroid therapy for drug-induced acute interstitial nephritis, using renal function after six months as the outcome [4]. Table 3 summarizes the previously published cases of renal dysfunction associated with Beni-koji tablets, with the exclusion of one case of recurrence. Among 26 cases from 15 case reports [5-19], fewer than one-third (eight patients [13-19]) were followed for more than four months, and follow-up beyond six months was uncommon [13,15,17-19]. Furthermore, recovery to normal eGFR (≥60 mL/minute/1.73 m²) at the final appointment was reported for six patients [8-11,15,17]. Regarding treatment, corticosteroid therapy tended to be administered to cases with more severe renal dysfunction or those with histologically confirmed severe tubulointerstitial injury. Similarly, the nationwide survey involved a relatively short observation period, ranging from several weeks to months, and only 13% of the patients attending the final appointment (13/100) were reported to have an eGFR ≥60 mL/minute/1.73 m² at the final appointment.

**Table 3:** Summary of the published cases of renal dysfunction associated with Beni-koji tablet intake. This table lists the clinical characteristics, kidney biopsy results, therapeutic interventions, and outcomes reported in previously published case reports. One case with recurrence was excluded. NA = not available; sCr = serum creatinine; eGFR = estimated glomerular filtration rate

Reference	Age (years)/Sex	Duration of Beni-koji tablet use	Kidney biopsy	Corticosteroid therapy	Baseline sCr (mg/dL)/eGFR (mL/minute/1.73 m²)	Peak sCr (mg/dL)/ eGFR (mL/min/1.73 m²)	sCr (mg/dL)/ eGFR (mL/minute/1.73 m²) at the last follow-up appointment	Approximate timing of the final follow-up appointment
Miyazaki et al. [5]	47/Female	7 months	Yes	Yes	1.09/44	4.70/NA	1.72/NA	4 weeks after admission
Uchiyama et al. [6]	56/Female	6 months	Yes	No	0.70/67.1	1.89/22.5	1.04/43.3	35 days after presentation
	56/Male	4 months	Yes	Yes	0.93/66.2	13.55/NA	1.69/NA	2 months after presentation
Takeuchi et al. [7]	66/Female	14 months	Yes	No	0.61/NA	1.74/NA	NA/NA (no full recovery)	3 months after presentation
	54/Male	2 years	Yes	No	0.84/NA	1.31/NA	NA/NA (no full recovery)	2 months after presentation
Koshida et al. [8]	42/Male	6 months	Yes	Yes	NA/NA	2.12/29	1.04/64	45 days after presentation
	83/Female	2 months	No	No	1.42/28	3.52/10	NA/NA (no full recovery)	2 months after discontinuation
Matsui-Hosoya et al. [9]	59/Female	7 months	Yes	No	0.65/71.6	2.32/17.7	0.96/46.5	86 days after admission
	48/Male	2 years	No	No	0.88/74.4	1.12/56.8	0.92/70.0	54 days after presentation
	47/Male	6 months	No	No	0.81/81.4	1.09/58.5	1.13/56.2	86 days after presentation
Maiguma et al. [10]	58/Female	6 weeks	Yes	Yes	NA/NA	2.75/NA	NA/NA	3 months after presentation
Yoshikawa et al. [11]	43/Male	1 year	Yes	No	0.9/74	2.11/NA	0.9/74	82 days after discontinuation
	54/Male	4 years	Yes	No	NA/NA	1.75/NA	1.3/46.3	110 days after discontinuation
Murata et al. [12]	73/Female	8 months	Yes	Yes	NA/NA	1.27/32.2	NA (no full recovery)	3 months after discontinuation
	53/Female	8 months	Yes	Yes	NA/NA	1.41/31.5	NA (no full recovery)	3 months after discontinuation
	55/Female	9 months	Yes	Yes	NA/NA	2.38/17.6	NA (no full recovery)	3 months after discontinuation
Chikasue et al. [13]	49/Female	2 weeks	Yes	Yes	0.84/56.8	21.9/1.6	1.66/26.8	160 days after admission
	55/Male	6 months	No	No	0.70/81.2	2.33/24.3	1.08/56.5	50 days after admission
	60/Female	7 months	No	No	0.62/76.2	3.71/10.5	1.11/39.5	95 days after admission
Abe et al. [14]	50/Male	7 months	Yes	No	NA/NA	3.99/13.9	1.88/31.6	4 months after discontinuation
Habuka et al. [15]	49/Female	18 months	Yes	No	NA/NA	1.5/35.2	0.74/NA	22 weeks after presentation
	68/Female	7 months	Yes	No	NA/NA	3.0/12.9	1.34/NA	26 weeks after presentation
Oda et al. [16]	62/Male	18 months	Yes	No	NA/NA	1.43/40.1	1.21/48.2	5 months after admission
Kamada et al. [17]	51/Female	2 months	Yes	Yes (5 days)	NA/NA	1.1/42.0	0.79/60	6 months after discontinuation
Iwamura et al. [18]	74/Male	10 weeks	Yes	Yes	0.95/59.9	1.21/45.8	1.12/49.8	7 months after discontinuation
Ushimaru et al. [19]	60/Female	35 days	Yes	No	0.57/NA	3.07/NA	0.85/NA	8 months after discontinuation

In the present study, the first patient ultimately developed chronic kidney disease (CKD), with an eGFR <60 mL/minute/1.73 m² that persisted for more than three months [20], whereas the second did not develop CKD. Given that CKD is associated with a high risk of cardiovascular disease and all-cause mortality [20], the difference in CKD status that we identified through the follow-up of the two patients for more than six months may be of clinical relevance.

The number of patients in whom Beni-koji tablet-associated renal dysfunction has been characterized remains limited, with 189 cases having been reported in the nationwide survey performed by the Japan Society of Nephrology [2] and 348 requiring hospitalization for renal disease treatment according to the Ministry of Health, Labour, and Welfare [1] because the associated renal dysfunction only began to be identified in late 2023 and the product recall occurred as recently as March 2024. Regarding pathogenic mechanisms, pulverulic acid and two other compounds (referred to as Compound Y and Compound Z in the Ministry of Health, Labour, and Welfare report) were identified from the contaminated lots associated with the reported cases. Furthermore, pulverulic acid was detected in cultures of blue mold (*Penicillium adametzioides*) obtained from the manufacturing facility, and experimental administration of pulverulic acid to rats resulted in proximal tubular degeneration and necrosis. Taken together, these findings suggest that pulverulic acid may be a potential cause of Beni-koji-associated renal dysfunction. However, the underlying pathophysiology, effective treatments, and the typical course of renal function recovery have not yet been fully elucidated.

These cases suggest that the recovery period in Beni-koji-associated renal dysfunction is variable and may be prolonged. Therefore, long-term follow-up is important to identify those at risk for progression to CKD and optimize their clinical management.

## Conclusions

A study of these two cases has shown that renal recovery after Beni-koji-associated renal dysfunction can follow different courses, ranging from incomplete improvement to recovery to baseline. It also implies that renal function may change over several months, such that patients may show a delayed improvement or long-term residual impairment. Taken together, these findings provide case-based insight into the heterogeneous nature of renal recovery.
